# Systematic Review of Mendelian Randomization Studies on *Helicobacter pylori*–Associated Health Outcomes

**DOI:** 10.1155/ipid/7756944

**Published:** 2025-10-22

**Authors:** Ikuko Kato, Federico Canzian, Cosmeri Rizzato, Antonia Rodriguez, Javier Torres

**Affiliations:** ^1^Department of Oncology, Wayne State University School of Medicine, Detroit, Michigan, USA; ^2^Department of Pathology, Wayne State University School of Medicine, Detroit, Michigan, USA; ^3^Population Sciences in the Pacific Program, University of Hawaii Cancer Center, Honolulu, Hawaii, USA; ^4^Genomic Epidemiology Group, German Cancer Research Center (DKFZ), Heidelberg, Germany; ^5^Department of Biology, University of Pisa, Pisa, Italy; ^6^Research Group in Cancer Biology, National Cancer Institute, Bogota, Colombia; ^7^Infectious and Parasitic Diseases Research Unit, XXI Century National Medical Center, The Mexican Institute of Social Security, Mexico City, Mexico

**Keywords:** *Helicobacter pylori*, Mendelian randomization, serology, systematic review

## Abstract

**Background:**

*Helicobacter pylori* (HP) infection has been linked to nearly 90 different health conditions, including gastric malignant and premalignant lesions. Recently, Mendelian randomization (MR) has gained popularity to overcome limitations in observational studies. This review aims to compile MR studies on the causal relationship between HP infection and health outcomes, systematically assess the quality of individual studies, evaluate the overall evidence in comparison with other existing data, and identify common strengths and weaknesses in order to guide future research directions on HP-associated health outcomes.

**Materials and Methods:**

Eligible studies were identified from the two major biomedical literature databases, PubMed and Embase. After removing overlaps, we found 33 unique records published by July 10, 2024. Among those, 16 were qualified for full-text review as original research papers presenting MR analysis with HP infection as the primary exposure of interest.

**Results:**

Among the 16 studies, one was one-sample MR study and the rest 2-sample MR studies. All except one were conducted on individuals of European descent. Health outcomes studied include four metabolic conditions, four cardiovascular diseases (CVDs), five gastrointestinal conditions, and three other miscellaneous conditions. All used genome-wide association study (GWAS) data on HP serology to select instrumental variants, ranging from 1 to 84. Nine out of 16 studies concluded that HP had a causal association with the disease of their interest. However, not all papers fully examined whether MR key assumptions were met and there are inaccurate descriptions and misinterpretations in many papers. In addition, there are inconsistencies between studies, depending on the choice of exposure GWAS from which instrumental variants were chosen.

**Conclusions:**

Overall, published MR studies concerning HP infection and various health outcomes to date are limited in their quality/integrity. The results from HP eradication trials completed or in progress may help address the causality of some of the health outcomes with high incidence rates. In addition, MR studies in non-European populations with higher HP prevalence are warranted.

## 1. Introduction

*Helicobacter pylori* (*HP*) is a Gram-negative, microaerophilic bacterium of the phylum Campylobacterota/Epsilonproteobacteria that colonizes the human stomach, which was discovered by Marshall and Warren in 1983 [1]. It is estimated that roughly half of the world's population is infected with this bacterium [2, 3], making it one of the most common human infectious agents worldwide. A recent updated meta-analysis of 1748 studies from 111 countries confirmed the high global prevalence of HP at 44%, despite a declining trend [4]. The prevalence of HP varies geographically [4] and is influenced by socioeconomic status (SES), smoking, and diet [5–7].

Significant advances have been made over the past 40 years in understanding the pathophysiology of HP. It has been causally linked to the development of gastric adenocarcinoma, to mucosa-associated lymphoid tissue (MALT) lymphoma, and to other nonmalignant gastroduodenal conditions, such as peptic ulcer, chronic gastritis, and intestinal metaplasia [8, 9]. In 1994, the International Agency for Research on Cancer/World Health Organization (IARC/WHO), classified HP as a Class 1 carcinogen, the only bacterium with this classification [8]. Furthermore, HP infection has been linked to as many as 90 different health conditions, which include both protective and detrimental associations [10]. However, the vast majority of the meta-analyses were poorly executed, with only 6 health outcomes having moderate quality evidence [10], making their causal inference challenging.

Even high-quality, observational studies are prone to various biases, confounding factors, and reverse causation [11], while exposures are usually measured at only one time point. In order to overcome these limitations and to infer causality, Mendelian randomization (MR), an approach that uses genetic variants associated with changes in exposure as instrumental variables (IVs), has gained growing popularity. The genetic code is fixed at conception, randomly assigned from parents, and therefore, MR investigations can be interpreted as assessing the impact of long-term exposure and can address the causal association if properly executed [12].

A recent explosive increase in the number of MR studies is likely to be driven by widely implemented data sharing policies, the availability of many genome-wide association study (GWAS) summary statistics, and the development of bioinformatic tools. This has made the execution of two-sample MR studies, which are based on different datasets for the exposure and outcome, more accessible to nonstatisticians [13].

The exposure, HP infection, can be measured by several diagnostic tests [14] with different pros and cons. Serology to detect IgG antibodies against various HP antigens is minimally invasive and least labor-intensive and thus commonly used in large epidemiological studies; however, this test has a limitation in distinguishing past and active infection. Other methods, such as breath test, stool antigen test, and endoscopic biopsies, can be used to detect active infection and to monitor eradication [14], although they present varied sensitivity and specificity. Choosing the right diagnostic test is critical in MR studies where HP infection represents the exposure variable.

This review aims to compile MR studies on the causal relationship between HP infection and health outcomes; systematically assess the quality of individual studies; evaluate the overall evidence in comparison with other existing data, such as meta-analyses; and identify common strengths and weaknesses in order to guide future research directions on HP-associated health outcomes.

## 2. Methods

The two major biomedical publication databases, PubMed and Embase, were used to identify eligible studies. We applied the same search strategy to both databases, using “Mendelian randomization” AND “*Helicobacter pylori*” in any field. We did not use any filters to limit publications, and the search was limited to July 10, 2024. The inclusion criteria were original research, being an HP infection as the primary exposure of interest, and presenting the results from MR analysis. Both one- and two-sample studies were included. Preprints were not included. To select eligible studies, we reviewed the full text as well as all online materials. Screening and data collection were carried out by a single reviewer (I.K.) with consultation with another reviewer (A.R.). Additional information concerning individual IVs used in eligible studies was obtained by FUMA, an integrative web-based platform or functional annotation of GWAS results [15].

A total of 24 publications from PubMed and 29 publications from Embase were identified. Among these, 20 were overlapped, leaving 33 unique records. Among these 33, 17 were excluded for the following reasons: 6 were nonoriginal research (e.g., editorial, review, etc.); 9 did not use HP as main exposure, 1 was a non-MR analysis, and 1 was a preprint of one of the publications that was left in Embase. The remaining 16 were included in the full review. For each eligible publication, we assessed the type of study (one- vs. two-sample), definition of exposure, outcome characteristics, instrumental variant selection criteria, the three key assumptions for MR studies (relevance, independence, and exclusion) [13], statistical methods used, main results, and authors' conclusion. We did not review the reverse-association analysis when bidirectional MR [11] was performed in the eligible studies, because HP acquisition primarily occurs in early childhood [8, 9], and therefore, it is unlikely that, in adulthood, a health condition may cause HP infection.

## 3. Results

### 3.1. Study Design

Among the 16 MR studies identified, only one was a one-sample MR study [16], while the remaining 15 were two-sample MR studies. Nine of the 16 studies performed bidirectional analyses and six also examined potential mediators besides major health outcomes to test vertical pleiotropy, including closely related conditions (such as gastritis, ulcers, heart failure, and hypertension) and/or biomarkers (such as circulating lipids, glucose, and cytokines). All studies were conducted in individuals of European descent, except one consortium study on coronary heart disease (CHD) that included mixed races [17].

### 3.2. Exposure Definition

All 16 studies chose serology to assess exposure to HP infection and used one of the three published GWASs on HP serology (Table 1) or their combinations. Two of the studies were based on HP whole-cell antigens, while the other was based on 6 specific HP antigens. The first study published in JAMA [18] utilized a commercial enzyme-linked immunosorbent assay (ELISA) kit from Finland, Pyloriset EIA-G III, which employed HP whole-cell acid extract as the antigen. The manufacturer recommended a cutoff for seropositivity of 20 U/mL, with a reported 98% sensitivity and 58% specificity. However, to improve specificity and to reduce false positives, the authors revised the cutoff with the upper 25% of the IgG titer distribution of each cohort included in the study. Three population-based cohorts of middle-aged men and women from the Netherlands and Germany were used for the GWAS to identify genetic loci associated with HP infection, while one cohort was used for a validation study to test serology data against the HP stool antigen test. This validation study demonstrated a high correlation between serum IgG antibody and stool antigen levels (Spearman correlation 0.59). The main GWAS revealed two loci (*p* < 5 × 10^−8^) strongly associated with the binary status of seropositivity. One was mapped to chromosome (Chr) 4p14 near *TLR10,* and the other to Chr 1q23.3 near *FCGR2A*, with minor allele frequency (MAF) of 26% and 16%, respectively. The single nucleotide polymorphism (SNP) on chromosome 4p14 was also reported to be associated with differential gene expression of *TLR1,* which is located within the same locus as *TLR10* [18]. It was also estimated that the two variants from this study explained 0.5% of HP seropositivity [16].

The study by Chong et al. also analyzed HP serology data obtained by in-house ELISA using commercially available HP whole-cell lysis antigens of strain 43504 from Australia (Meridian Life Science) [19]. This was part of a larger study to investigate the prevalence of antibodies against 12 common infectious agents and two caseins in children from 5 to 15 years old enrolled in the Avon Longitudinal Study of Parents and Children [20]. The data available for this GWAS were from 7-year-old children. The number of children tested for each antibody varied with the types of infectious agents, and there were 4683 children tested for HP [19]. The study did not find a clear cutoff to define seropositivity for HP, and thus, the data were treated as continuous values. Applying the *p* < 5 × 10^−8^ criterion, the authors found one variant mapping to an intergenic region of Chr 6 between *HLA-DOB1* and *MTCO3P*.

Butler-Laporte et al. used a different approach to measure various HP IgG antibodies, i.e., multiplex Luminex assay, which was a GST capture ELISA combined with fluorescent bead technology [21]. This was part of a phenotype determination effort within UK Biobank concerning infection with 20 common microorganisms in subsamples [22]. HP antibodies against six different antigens, CagA, VacA, OMP, GroEL, Catalase, and UreA, were quantified. The cutoff value for seropositivity to each antibody was determined with the use of additional reference seronegative sera. For the GWAS summary dataset for 8735 individuals of European descent, seropositivity (binary) was defined as positivity to two or more HP antigens, except for CagA that is present only in Type I HP strains. Quantitative antibody data for the six individual HP antigens were included in GWAS summary data only if the values were above the seropositivity cutoff, since the values below the cutoff values were likely to represent nonspecific reactions. Thus, the sample sizes available for each antigen varied from 985 (CagA) to 2716 (GroEL). Applying the *p* < 5 × 10^−8^ criterion, the authors found two variants associated with lower seroreactivities to OMP and UreA. Both variants were mapped to Chr 6 close to *HLADQB1* for OMP and *RP11-439H9.1* for UreA.

### 3.3. Health Outcomes Studied (Table 2)

As shown in Table 2, a broad range of health outcomes were investigated in these 16 studies, which comprised four metabolic conditions (obesity [16], nonalcoholic fatty liver disease (NAFLD) [23], Type 2 diabetes (T2DM) [24], and osteoporosis [25]), four cardiovascular diseases (CHD [17], myocardial infarction (MI) [26], stroke [27], and atherosclerosis (AS) [28]), five gastrointestinal conditions (including colorectal cancer (CRC) [29], gastroesophageal reflux disease (GERD) [30], irritable bowel syndrome (IBS) [31], inflammatory bowel disease (IBD) [32], and eosinophilic esophagitis (EOE) [33]), and three others (pregnancy-associated complications [34], glaucoma [35], and IgA nephropathy (IgAN) [36]). Some of these outcomes were further divided into subclasses within the main outcome (e.g., type of CHD, stroke, AS, CRC, and glaucoma).

Most outcomes were binary traits (presence or absence), while a few related to obesity and pregnancy complications were continuous. Except for the one-sample study [16], the sources of these outcomes were either big consortia/cohorts/case–control studies of specific health outcomes or big biobanks from UK (UK Biobank) and Finland (FinnGen). About half of the studies (*N* = 7) included more than one source of GWAS data for the outcome, either combinations of data from two biobanks, consortia, big cohort, or case–control studies. The number of cases for the binary traits was generally over 1000, with few exceptions, i.e., cerebral AS (*N* = 150), placental abruption (*N* = 294), and rectal cancer (*N* = 375). Outcome definition varied widely. Consortium studies generally had strict inclusion criteria for specific medical conditions. For example, histological confirmation was required for NAFLD and EOE [23, 33] and combinations of symptoms and radiological or endoscopic or histological findings for IBD [32]. However, diagnostic criteria used in huge consortia that comprised a mix of prospective cohorts and cross-sectional/case–control studies, e.g., those for CHD [17, 26] and stroke [27], were variable depending on the protocols in individual studies. Outcomes from biobanks were generally derived from record linkage with various electronic health records, but the UK biobank offered several different sets of outcomes besides consolidated hospital discharge diagnoses. Specifically, self-reported history was used in the study by Wang et al. for IBS [31] and self-reported history of osteoporosis was used in the study by Zhang et al. [25]. Chen et al. used the secondary diagnostic codes of inpatient records to study GERD [30]. These authors, however, did not provide the rationale for choosing these specific datasets with less reliable diagnoses. Luo et al. did not specify what level of diagnosis within UK biobank was used in their study for CRC. The controls derived from the biobanks were not the same as in controls selected for typical case–control studies, but comprised the participants who did not develop specific outcomes of interest. Controls in consortium studies also varied from hospital, population, or friend/family controls to those from other consortia, depending on the choice of individual studies that participated. Most studies were conducted on adult patient populations, except for EOE that was limited to pediatric patients [33] and one of the IBD outcome cohorts that included both pediatric and adult-onset cases [32].

### 3.4. Instrumental Variant Selection Criteria

Instrumental variants were first selected based on *p*-values reported in the original GWAS for HP serology described above. The original selection criteria, *p* < 5 × 10^−8^, MAF > 1% and variants with the lowest P within the loci, were used in the studies that derived instrumental variants from the HP GWAS by Mayerle [18], as this GWAS was not in publicly available GWAS datasets. In MR studies based on two other HP serology GWASs [19, 22], the most common *p*-value cutoff was *p* < 5 × 10^−6^, while three studies adopted less stringent cutoffs, *p* < 5 × 10^−5^ [24, 34] or *p* < 1 × 10^−5^ [36]. Studies with the higher cutoff claimed to have removed variants with weaker association based on F-values, but there were no data reported as to how many SNPs were actually eliminated. Most of the studies also implemented removal of variants in linkage disequilibrium (LD), clumping variants with *R*^2^ = 0.001 threshold and 10,000 kb distance. Some studies further applied additional selection based on MAF (≥ 0.01) [34], or exclusion of ambiguous or palindromic SNP [29, 32, 34, 36], SNPs with intermediate frequencies [29, 32], outliers [28], and SNPs associated with potential confounders [27, 30] (Table 3). The ultimate numbers of SNPs used in the analyses were further limited by their availability in the outcome GWASs, ranging from 1 to 84 (Table 2). We compiled SNPs used in these 16 studies downloading also all online materials (if any).  Table 4 presents the location and type of SNPs, nearest genes, and measures for potential functions for the 89 SNPs on 84 genomic loci with *p* < 5 × 10^−6^. There were 4 SNPs with *p* < 5 × 10^−8^, 10 with P between 5 × 10^−7^ and 10^−8^, and the rest with *P* ≥ 5 × 10^−7^. Surprisingly, there was no single overlap among instrumental SNPs selected based on different HP serological tests and none of the SNPs were in LD with any of the others in the list (*r*^2^ < 0.2).

### 3.5. Tests for the Key MR Assumptions and Statistical Methods

There are three key assumptions to fulfill to carry out valid MR studies. The first assumption concerns relevance that ensures variants are reliably associated with the exposure [13]. All studies demonstrated that the selected instrumental SNPs had sufficiently strong association with the HP serology measures based on F-statistic of 10 or greater, which has been generally accepted to rule out weak instruments [13]. However, there are marked discrepancies in the estimate of the strength of selected instruments due to the two different formulas to calculate F-statistics, one based on *R*-squares that indicates the proportion of the variance in the phenotype explained by specific variants [37], while the other is based on beta^2^/standard error (SE)^2^ in the exposure GWAS. For example, the SNP mapped to Chr 4p14 near *TLR10* that showed the strongest association [18] was reported to have F-statistics of 548 [38], 478 [23], 236 [17], and 78 [24]. The first three values appear to be erroneous due to inappropriate calculation of R-squares from the logistic regression. Similar systematic discrepancies on F-statistic values have also been noted for instrumental variants selected from the Avon cohort [26, 34].

The second key assumption concerns independence that ensures no unmeasured confounders explaining the association between instrumental variants and outcome. To meet this assumption, three studies [29, 30, 36] implemented the removal of variants associated with potential confounders through search in PhenoScanner, a curated database of publicly available results from > 5000 large-scale GWAS to catalogue of human genotype–phenotype associations [12], and LDtrait, a web tool for finding germline variation associated with multiple traits. Details of search criteria or the number of variants removed were not reported in studies on GERD or CRC, but Jing described a search for variants associated with a wide range of potential confounders, e.g., age, weight, hypertension, proteinuria, dental caries, periodontitis, tonsillitis, and other infections, including respiratory pathogens (e.g., *Mycoplasma pneumoniae*, herpes virus, influenza) and gut microbiota [36]. Guo et al. described [27] the removal of variants with strong associations (undefined) with alcohol, smoking, body mass index (BMI), diabetes, and educational level. Again, there were no details concerning the definition of strong association or the number of variants removed. Zhu et al. implemented a UK Biobank GWAS data search for variants associated with confounders (education attainment, household income, and deprivation), which may affect HP acquisition. Instead of removing those variants from the main MR analysis, the authors executed multivariate MR analyses to control the effects of these confounders [33]. There was no information as to how many potential confounders were included in these statistical models.

The third key assumption is exclusion restriction that ensures that IVs are linked to the outcome only though the exposure of interest [13], ruling out horizontal pleiotropy. To meet this assumption, IVs should not be linked to the outcome through other independent biological mechanisms not on the direct vertical pathway of the exposure. Several statistical methods have been developed to detect horizontal pleiotropy. These include MR-Egger intercept, Cochran Q statistics, and MR-PRESSO global test [39–41]. The most commonly used were the first two, while some additionally or alternatively used MR-PRESSO (Table 3). Although no studies reported the presence of significant horizontal pleiotropy, some disregarded positive results from these tests. For example, in the MR study for T2DM, heterogeneity was present for anti-GroEL and anti-UreA antibodies (*p* < 0.01) [24]. In the study for CRC, the *p*-value from Q statistics for the association between colon cancer and seropositivity was 0.03 [29]. Furthermore, no studies reported whether they eliminated IVs that had a direct association with the outcome at the genome-wide level (*p* <  5 × 10^−8^) to fulfill this key assumption.

### 3.6. Main Results and Authors' Conclusion

Main causal estimates based on the inverse variance weighting (IVW) method were reported in all studies. As shown in Table 2, the summary odds ratios (ORs) for primary endpoints in individual studies clustered around 1.00, ranging from 0.325 to 1.23, except some skewed estimates (OR > 7) from studies with small case count, e.g., cerebral AS [28] or small size of outcome cohort, e.g., intracerebral hemorrhage [27]. Many of the statistically significant ORs showed an association of the magnitude close to 1.1, but the ORs that were almost 1.00, such as 1.001 (GERD), 1.002 (osteoporosis), and 1.03 (T2DM), were also found statistically significant owing to their large numbers of cases and controls included in the outcome GWAS. Nine out of the 16 studies reported statistically significant associations at the conventional level of *p*-values between selected IVs and the major endpoints (not including mediators), and the authors concluded that HP infection or specific types of antibodies against HP were causally associated with the primary diseases of interest. These include CHD [17], MI [26], osteoporosis [25], some of the pregnancy complications (preeclampsia–eclampsia and premature rupture of membranes) [34], T2DM [24], GERD [30], AS [28], stroke [27], and EOE [33]. The rest of 7 studies on CRC [29], IBS [31], IBD [32], obesity [16], glaucoma [35], NAFLD [23], and IgAN [36] found no association, and the majority concluded that there was no evidence or no genetic evidence for causal association. One study on IBD [32] referred to no direct correlation with HP infection, while the conclusion was more conservative in the IBS study, as mentioned “may not be causally associated” [31].

It is important to note that multiple primary outcomes (subtypes of diseases) and/or multiple sets of exposure IVs were included in the analysis in most studies and that the strength of the observed statistically significant associations at the conventional level was often insufficient to sustain through adjustment for multiple comparisons. Zhu et al. reported Benjamini–Hochberg corrected *p*-values for multiple testing in their study for EOE [33] and Li et al. presented Bonferroni-corrected *p*-value thresholds for their study on CHD [17]. 0.325 was the lowest reported OR for the association between EOE and HP seropositivity; however, this result was highly questionable because the risk estimates from individual IVs shown in their online data were not consistent with it [33]. In addition, the conclusion by Li et al. [17] that “HP infection exerts a causal effect on CHD incidence, mediated by BMI” does not seem well justified as the primary outcomes (except angina) did not show a significant association with IV, while BMI was strongly associated with IV.

## 4. Discussion

Prior to MR, evidence was present from meta-analyses to support potential associations between HP infection and health outcomes examined in the individual MR studies [10, 42, 43], except for IgAN. There were weak clinical data to support a potential causative role of HP in IgAN, while HP infection was inversely associated with the risk of end-stage renal disease in a meta-analysis [10]. The published meta-analyses generally supported the positive (detrimental) association between HP infection and the health outcomes, i.e., obesity, cardiovascular diseases, osteoporosis, T2DM, NAFLD, pregnancy complications, glaucoma, CRC, and IBS [10, 42–44], while meta-analyses for a minority of the outcomes suggested that HP might be protective against IBD, EOE, and GERD [10, 45]. The authors argued that despite the published data supporting the association, there were inconsistencies among observations, potential residual effects from confounding factors, which were not ruled out, and no clear biological mechanisms that explain the associations. That was the common rationale to conduct MR analyses.

There are several critical issues in interpreting the results of these MR studies. The major concern was the validity of IVs to determine exposure to HP. Two of the HP serology GWASs defined binary HP seropositivity status. The first study by Mayerle et al. defines seropositivity by the relative distribution of antibody titers, not using known negative samples and without consideration of cohort characteristics [18]. This would have led to reduced sensitivity and increased false negatives, particularly in older birth cohorts because they are more likely to have been exposed to HP [46, 47] and because antibody response declines with age. In the study by Butler–Laporte, seropositivity was determined by the number of positive antibodies against 5 HP antigens, excluding CagA [22]. Whereas the positive cutoff for each type of antibody was established using known seronegative samples [21], the accuracy of overall seropositivity based on the number of positive antibodies was not reported.

All six HP antigens to which antibodies were analyzed in UK biobank samples [22] are known HP virulence factors required for colonization of the stomach and have robust antigenicity [21]. However, four of these proteins are not HP-specific and can be produced by other bacteria, leading to possible cross reactivity in the assay (false-positive results). Importantly, because GWAS summary data for individual antibody levels from Luminex assays included only values above the positive cutoff, participants who were negative to specific antibodies were excluded. Consequently, these data do not help to address genetic substitutability to acquisition of HP, but may instead help to identify genetic variabilities in adaptive immunological responses in producing specific antibodies among those exposed to HP. On the other hand, IVs associated with seropositivity may reflect the probability of HP exposure, which can be affected by living conditions and SES, but also the innate immune response that acts as the first line of defense to foreign pathogens, as well as the specific adaptive immunity that leads to antibody production against pathogen proteins. This is consistent with the variant loci found in the study by Mayerle et al., i.e., *TLR10/TLR1* and *FCGR2A* [18].

A more serious concern is the IVs from 7-year-old children in the Avon cohort, since the accuracy of serology for HP infection in young children (i.e., < 10 years), whose immune function is not fully developed, has been reported to be lower than other diagnostic tests [48, 49]. Because HP prevalence in children in high-income countries is estimated to be about 20% [50], a large portion of the continuous antibody data are likely to reflect unspecific reactions in HP-negative children. Moreover, the validity of 2-sample MR studies where exposure and outcome cohorts include individuals with different age ranges is questionable.

Serological tests to measure antibodies to HP antigens have been used in epidemiological studies under the premise that persistent antigen stimulation elicits an antibody response lasting for years. Accordingly, antibodies serve as markers for current or past HP infection. Thus, the statements suggesting that antibodies themselves are actually harmful and responsible for the causation of specific health conditions (Table 3) are misleading [27, 28, 33, 44]. An exception may be autoimmune mechanism, i.e., molecular mimicry, induced by HP infection [51]. Specifically, anti-CagA antibodies cross-react with human trophoblast cells, causing functional impairment and placental damage, which may increase the risk of preeclampsia [52]. In addition, anti-CagA antibodies have been demonstrated to interact with vascular antigens of 160 and 180 kDa, which are present in different parts of smooth muscle cells and endothelial cells of normal and atherosclerotic vessels [53]. Antibodies against HP urease have also been suggested to recognize the IKEDV motif in the CC chemokine receptor-like 1 (CCRL1), which complement-dependent tissue destruction and inflammatory response that contribute to AS and cardiovascular disease [51]. Likewise, antibody against HP GroEL (human Hsp60 homolog) has been postulated to promote CHD development through antigenic mimicry and complement-dependent cell damage, as the antiurease antibodies [51]. Among the 6 types of antibodies against specific HP antigens, VacA antibody was most often associated with health outcomes, i.e., coronary AS [28] and stroke [35]. It was not clear if this was due to generally larger number of IVs used for VacA compared with other HP antigens or due to higher antibody variabilities reflecting infection with HP *VacA* sequence variants.

Furthermore, it is imperative to note that some of the health outcomes (including potential mediators) were studied more than once with different sets of IVs from different GWASs. These include BMI, fasting blood sugar, and low-density lipoprotein (LDL) cholesterol and the results were highly inconsistent, with some showing opposite directions of the association (Table 2). This raises a serious concern about the reproducibility of these MR analyses.

Den Hollander reported that 0.5% of HP seropositivity was explained by the two most stringent variants found in their cohorts [16]. This fraction was indeed equivalent to those for other external risk factors that have been studied by MR, e.g., alcohol intake, smoking intensity, and coffee consumption (1% or lower), while those for host-related risk factors, such as height, bitter-taste sensitivity, and total cholesterol level, were much higher (13%–43%) [54]. As shown in Table 4, IVs selected by HP serology include a cluster of variants in the major histocompatibility complex (MHC) class 2 locus, which are also classified as gene expression quantitative trait loci (eQTL). While this locus plays a crucial role in antigen presentation and adaptive immunity, it is not clear if these associations were HP antigen-specific. In addition, while the selection criteria for the *p*-values were rather lenient, highly stringent criteria were used throughout these MR studies to exclude variants in LD. Thus, whereas this eliminates redundancy in IVs, it is possible that other potentially functionally important variants were not tested for the outcomes, because they were in LD with the SNPs selected as IVs, and therefore were discarded from analyses.

Most studies employed sets of bioinformatic analyses to detect horizontal pleiotropy, heterogeneity, and outliers for the exclusion restriction, as well as carried out sensitivity analysis using multiple statistical methods to summarize the effects of individual variants to test the robustness of the results. However, only a limited number of studies addressed the independence assumption concerning the association with potential confounders. Given a number of factors associated with HP acquisition [5–7] and shared risk factors among many chronic diseases, such as cardiovascular disease and cancer [55], this poses a serious concern in MR studies that do not test for this assumption. When it was tested, PhenoScanner [12] was the most commonly used tool to search variants associated with potential confounders. Confounders, by definition, must be correlated with both exposure and outcome; however, the definitions of potential confounders searched varied from study to study [27, 33, 36], and otherwise, no specifics were reported [29, 30].

A further disturbing finding was the lack of attention to the quality of outcome datasets. Implementation of data sharing policy has widened public access to large GWAS data, but the structure of those huge biobanks and consortia is often very complex and expertise/experience is required to retrieve the appropriate datasets for specific phenotypes. Moreover, in addition to the erroneous calculation of F-statistics discussed above, there were many seemingly erroneous data. For example, the source of the first HP GWAS by Mayerle et al. [18] was quoted wrongly as the GWAS dataset from Chong et al. [19] by two studies [17, 29]; alternatively, Chong's GWAS data were inaccurately described as the case–control data [23]; HP seropositivity determined by multiplex Luminex assays [22] was described as continuous data in one study [27]; IV selection criteria were described as *p* < 5 × 10^−8^ in the methods, but actual analyses included those with *p* < 5 × 10^−6^ [25]; a scatter plot was replaced by a Forest plot from completely different data in the study by Yang et al. [32]; and online data in the AS study listed a mixture of original beta and ORs for the OR [28]. Moreover, Guo et al. emphasized the importance of VacA-positive HP infection for stroke, despite the fact that VacA is present universally in HP [35]. These errors could have been prevented if the authors had implemented more rigorous research practices or included investigators with relevant expertise. Equally, this may represent failures in peer review, which often has to be completed within a short period of time. The quality of published work should be given more priority than accelerated publications.

The reported risk estimates from these MR studies, which represent an increase in outcome risk per allele, were mostly modest, although limitations in estimating the effect size in MR studies have been recognized [56]. Excluding the two studies discussed above for questionable conclusions [17, 33], the results from 6 other MR studies that yielded a significant positive association [24–26, 28, 34, 35] were consistent with those from prior meta-analyses in terms of the direction of the association. However, the magnitude of the associations was much weaker compared with those from meta-analyses of observational studies [10, 42, 43]. On the contrary, a positive association was found for GERD by MR analysis [30], while meta-analysis was in support for the inverse association [45]. Because the risk estimate from this MR study was negligible (OR = 1.001) with *p*-value of 0.043, it should rather be considered as null association. Biological and methodological knowledge about the relations between exposures and outcomes is critical for interpreting results of MR studies [13]. While negative results from MR studies may suggest residual confounding in traditional observational studies, given the small fraction of exposure explained by IVs, potential causal involvement should not be completely ruled out if strong biological data support the link. On the other hand, given imperfect knowledge about genetic pleiotropy and intrinsic uncertainty in IV assumptions [56], a positive MR result alone may not necessarily suffice to establish a causal association, even if rigorous assessment for MR key assumptions and a range of sensitivity analyses to confirm the robustness of the association were carried out. Some MR studies included potential mediators to shed light on mechanistic pathways. C-reactive protein (CRP), high-density lipoprotein (HDL) cholesterol, and blood glucose levels were suggested to be potential mediators for stroke [27], MI [26], and T2DM [24], respectively. While the proinflammatory property of HP was speculated to be the link between HP and these mediators for stroke and MI, HP-induced alterations in appetite/energy expenditure controlling hormones, leptin, and ghrelin [57], which are secreted from the gastric mucosa, have been proposed to be the underlying mechanism for insulin resistance.

Finally, despite the complete overlap of the eligible publications between the two databases searched, it is possible that we missed other studies published in journals not indexed in either database. Those reports are more likely to include studies with lower quality and unfunded studies, and thus, it is unlikely to change the conclusion of this review.

## 5. Conclusions

Overall, published MR studies concerning HP infection and various health outcomes to date are limited in their quality/integrity with erroneous data or interpretation, as well as in uncertainty in fulfillment of the key MR assumptions and heterogeneity in the results between and within studies on similar/subclasses of health outcomes. Since MR studies sit at the interface of intervention and observational studies [13], the results from HP eradication trials may help address the causality of some of the health outcomes with high incidence rates, while more reliable and comprehensive HP exposure GWAS data may help gain reproducible MR results. Finally, MR studies to date have been exclusively conducted in individuals of European descent, despite the fact that HP is more prevalent in non-white populations. Thus, MR studies in non-European populations are equally warranted.

## Figures and Tables

**Table 1:** Description of three GWAS studies for HP serology.

Study	Author, year	Study population	HP exposure assessment	SNP typed/imputed
1	Mayerle, 2013	Population-based cohorts of middle-aged adults from the Netherlands (two cohorts, mean age 69/65) and Germany (mean age 50) (total *N* = 10,938)	Commercial ELISA (Pyloriset, Orion, Finland) using whole-cell acid extract antigen. Seropositivity (binary) was defined as upper 25% titers in each cohort	∼2.5 M

2	Chong, 2021	7-year-old British children enrolled in the Avon Longitudinal Study of Parents and Children (*N* = 4683)	In-house ELISA using commercial whole-cell lysis antigen. Antibody titers (continuous) were in normalized value, no cutoff for seropositivity established	7,247,045

3	Butler-Laporte, 2020	Europeans from 9695 UK Biobank participants age 40–69 tested for 20 common pathogens	Multiplex Luminex assay to detect antibodies against 6 HP antigens, CagA, VacA, OMP, UreA, Catalase, and GroEL: Overall seropositivity (binary) was defined as being positive to 2 or more antibodies, except CagA. Quantitative (continuous) data for antibody titers to each antigen were available only for samples with titers above positive cutoff	
		Seropositivity (*N* = 8735)		9,170,312
		CagA (*N* = 985)		9,165,056
		Catalase (*N* = 1558)		9,167,570
		GroEL (*N* = 2716)		9,172,299
		OMP (*N* = 2640)		9,167,440
		UreA (*N* = 2251)		9,170,248
		VacA (*N* = 1571)		9,178,635

**Table 2:** Summary results of 16 Mendelian randomization studies of HP infection for various health outcomes.

Author, year	One- vs two-sample MR	Health outcomes^∗^	Trait type	Study population (*n*)	No. of SNP typed/imputed	HP measures (reference)	No IV used in analysis	Summary causal effect estimates (*p* value)/per allele or polygenic score (based on IVW/Wald ratio)
*Metabolic conditions*								
den Hollander, 2017	1	**Body mass index (BMI)**	Continuous	Population-based cohorts of middle-aged adults from the Netherlands (mean age 69) and Germany (mean age 50) (total *N* = 11,690)	∼2.5M	HP seropositivity [18]	2	Beta = −0.06 (*p*=0.31)
		**BMI category (6 levels)**	Ordinal					Beta = −0.01(*p*=0.37)
		**Obesity (BMI ≥ 30)**	Binary					OR = 0.98 (*p*=0.47)

Liu, 2022	2	**Nonalcoholic fatty liver disease (NAFLD)**	Binary	1483 histologically confirmed cases from EPoS consortium and 17,781 population controls from other consortia	7,411,923	HP seropositivity [18]	1	OR = 0.755 (*p*=0.308)
				894 cases identified by health administrative records and 217,898 controls from FinnGen Biobank	16,380,466		2	OR = 1.048 (*p*=0.759)
		BMI (SD)	Continuous	454,884 UK biobank participants	9,851,867		1	OR = 1.022 (*p*=0.0015)
		Fasting blood glucose (SD)	Continuous	58,074 participants in 29 studies included in MAGIC consortium	2,599,409		2	OR = 1.006 (*p*=0.510)
		Triglycerides (SD)	Continuous	441,016 from UK Biobank	12,321,875		2	OR = 1.005 (*p*=0.409)
		HDL (SD)	Continuous	403,943 from UK Biobank	12,321,875		2	OR = 0.994 (*p*=0.788)
		LDL (SD)	Continuous	440,546 from UK Biobank	12,321,875		2	OR = 1.003 (*p*=0.514)

Zhang, 2024a	2	**Osteoporosis**	Binary	5266 self-reported osteoporosis cases and 331,893 controls from UK Biobank	1,08,94,596	HP ELISA titers [19]	12	OR = 1.002 (*p*=0.022)

Sun, 2024	2	**Type 2 diabetes (T2DM)**	Binary	24,133 cases, 183,185 controls from FinnGen Biobank	16,380,420	HP seropositivity [18]; CagA, Catalase, GroEL, OMP, UreA, and VacA antibody titers [22]	2/80/69/62/73/78/84 for seropositivity/CagA/Catalase/GroEL/OMP/UreA/VacA	ORs for HP seropositivity/CagA/Catalase/GroEL/OMP/UreA/VacA 1.10 (*p*=0.013)/1.00 (*p*=0.907)/1.00(*p*=0.846)/1.03 (*p*=0.028)/1.00(*p*=0.823)/0.99 (*p*=0.672)/1.00 (*p*=0.752)
		Blood glucose (FBS)	Continuous	400,458 participants in UK Biobank	4,218,897		15 for GroEL	1.01 (*p*=0.020)
		HbA1c	Continuous	467,601 participants in UK Biobank	13,586,180		2/65 for seropositivity/GroEL	OR for seropositivity/GroEL 0.93 (*p*=0.105)/1.02 (*p*=0.378)
		Class 3 obesity (BMI ≥ 40)	Binary	2896 cases, 47,468 controls from GIANT consortium, consisting of 51 various cohorts, case–control, and case series studies.	2,250,779		2/18 for seropositivity/GroEL	OR for seropositivity/GroEL 1.28 (*p*=0.012)/0.89(*p*=0.266)

*Cardiovascular diseases*								
Li, 2024	2	**Coronary heart disease (CHD)**	Binary	60,801 cases/123,504 controls from CARDIoGRAMplusC4D consortium, mix of case-control/cross-sectional and cohort studies worldwide (mixed race)	9,455,779	HP seropositivity [18]	2	OR = 0.99 (*p*=0.842)
				30,952 cases/187,840 controls from FinnGen Biobank	16,380,466		2	OR = 1.05 (*p*=0.178)
		**Angina pectoris**	Binary	18,168 cases/187,840 controls from FinnGen Biobank	16,380,426		2	OR = 1.11 (*p*=0.023)
		**Myocadiac infarction**	Binary	12,801 cases/187,840 controls from FinnGen Biobank	16,380,433		2	OR = 0.99 (*p*=0.889)
		Major cardiac events	Binary	21,012 cases/197,780 controls from FinnGen Biobank	16,380,466		2	OR = 1.02 (*p*=0.663)
				10,157 cases/351,037 controls from UK Biobank	13,295,130		2	OR = 1.00 (*p*=0.391)
		Cardiac deaths	Binary	7563 cases/211,229 controls from FinnGen Biobank	16,380,466		2	OR = 1.035(*p*=0.558)
		Arrythmia	Binary	2545 cases/460,388 controls from UK Biobank	9,851,867		1	OR = 1.00 (*p*=0.823)
		Herat attack	Binary	10,693 cases/451,187 controls from UK Biobank	9,851,867		1	OR = 1.00 (*p*=0.124)
		Stroke	Binary	7055 cases/454,825 controls from UK Biobank	9,851,867		1	OR = 1.00 (*p*=0.525)
		Herat failure	Binary	1405 cases/359,789 controls from UK Biobank	9,858,439		2	OR = 1.00(*p*=0.741)
		BMI (SD)	Continuous	454,884 UK Biobank participants	9,851,867		2	beta = 0.022 (*p*=0.001)
		Fasting blood glucose	Continuous	58,074 participants in 29 studies included in MAGIC consortium	2,599,409		2	beta = 0.006 (*p*=0.511)
		Triglycerides	Continuous	441,016 from UK Biobank	12,321,875		2	beta = 0.005 (*p*=0.409)
		HDL	Continuous	403,943 from UK Biobank	12,321,875		2	beta = −0.006 (*p*=0.788)
		LDL	Continuous	440,546 from UK Biobank	12,321,875		2	beta = 0.013 (*p*=0.515)

Wang, 2023	2	**Myocardial infarction (MI)**	Binary	14,825 cases/380970 controls from CARDIoGRAMplusC4D consortium, mix of case-control/cross-sectional and cohort studies (European)	10,290,368	HP ELISA titers [19]	11	OR = 1.10 (*p*=0.00007)
		Fasting blood glucose	Continuous	200,622 participants from MAGIC consortium and the Lifelines Cohort Study	31,008,728			beta = 0.005 (*p*=0.356)
		HbA1c	Continuous	45,734 from within-family consortium	9,696,819			beta = 0.013 (*p*=0.457)
		Triglycerides	Continuous	441,016 from UK Biobank	12,321,875			beta = 0.007 (*p*=0.207)
		HDL	Continuous	403,943 from UK Biobank	12,321,875			beta = −0.016 (*p*=0.002)
		LDL	Continuous	440,546 from UK Biobank	12,321,875			beta = −0.007 (*p*=0.222)
		BMI (SD)	Continuous	461,460 from UK Biobank	9,851,867			beta = −0.002 (*p*=0.656)
		Systolic blood pressure	Continuous	757,601 from UK Biobank and the International Consortium of Blood Pressure Genome-Wide Association Studies (ICBP)	7,088,083			beta = 0.034 (*p*=0.689)
		Diastolic blood pressure	Continuous	757,601 from UK Biobank and the International Consortium of Blood Pressure Genome-Wide Association Studies (ICBP)	7,160,619			beta = −0.015 (*p*=0.789)

Zhang, 2024b	2	**Coronary atherosclerosis (AS)**	Binary	28,598 cases/222,551 controls from FinnGen Biobank	16,962,023	HP seropositivity and CagA Catalase, OMP, and VacA antibody titers [22]	For HP seropositivity/CagA/Catalase/OMP/VacA 10/10/8/6/11	ORs for HP seropositivity/CagA/Catalase/OMP/VacA *p* > 0.05 if not specified 1.23/1.01/1.03/1.04/1.06 (*p*=0.02)
		**Cerebral atherosclerosis (AS)**	Binary	150 cases/241,202 controls from FinnGen Biobank			8/10/8/8/14	7.42/1.08/1.08/0.58/0.73
		**Peripheral atherosclerosis (AS)**	Binary	8393 cases/190,836 controls from FinnGen Biobank			10/10/8/6/14	0.93/1.05/0.97/1.33 (*p*=0.00026)/1.00
		**Other atherosclerosis (AS)**	Binary	8391 cases/244,907 controls from FinnGen Biobank			10/10/8/4/14	0.89/1.04/0.97/1.15/1.01

Guo, 2023	2	**All-cause stroke**	Binary	40,585 cases/406,111 controls from three consortia and 14 individual studies	8,211,693	HP seropositivity, OMP, UreA, Catalase, GroEL, CagA, and VacA antibody titers [22]	For seropositivity OMP, UreA, Catalase, GroEL, CagA, and VacA 11/8/10/7/4/11/15	ORs for HP seropositivity/OMP/UreA/Catalase/GroEL/CagA/VacA, *p* > 0.05 if not specified 0.87/1.00/1.00/1.01/1.08(*p*=0.041)/0.99/1.04(*p*=0.017)
		**Any ischemic stroke**	Binary	34,217 cases/406,111 controls from three consortia and 14 individual studies	8,296,492		11/8/10/7/4/11/15	0.85/0.99/0.99/1.01/1.05/0.98/1.03
		**Cardioembolic stroke**	Binary	7193 cases/204,570 controls from three consortia and 14 individual studies	8,271,294		11/8/9/7/4/11/15	0.71/0.97/0.98/1.00/1.13/0.93(*p*=0.036)/1.11(*p*=0.001)
		**Large artery stroke**	Binary	4373 cases/143,572 controls from three consortia and 14 individual studies	8,418,349		11/8/10/6/4/11/15	0.98/1.04/1.03/0.95/1.14/0.97/1.03
		**Small vessel stroke**	Binary	5386 cases/192,662 controls from three consortia and 14 individual studies	8,280,845		11/8/10/7/4/11/15	1.10/1.04/1.04/1.04/1.18/0.96/1.05
		**Intracerebral hemorrhage**	Binary	3223 cases/3725 controls from 6 hospital or population-based case–control studies in Europe and United States	5,358,102		1/2/6/3/1/2/6	7.61/1.55/1.08/0.86/1.05/0.92/1.18

*Gastrointestinal conditions*								
Luo, 2023	2	**Colorectal cancer**	Binary	4957 cases/304,197 controls from FinnGen Biobank	16,962,023	HP seropositivity [18]; CagA and VacA antibody titers [22]	2 seropositivity for all cancer, 13/15/15 vacA and 10/14/14 CagA for CRC/colon/rectal cancer	ORs for seropositivity/vacA/cagA 1.12 (*p*=0.08)/0.96 (*p*=0.25)/1.00(*p*=0.92)
		**Colon cancer**	Binary	1697 cases/359444 controls from UK Biobank	∼13.7M			1.00 (*p*=0.81)/1.00 (*p*=0.05)/1.00 (*p*=0.57)
		**Rectal cancer**	Binary	375 cases/360,766 controls from UK Biobank	∼13.7M			1.00(*p*=0.83)/1.00(*p*=0.05)/0.99 (*p*=0.19)

Chen, 2023	2	**Gastroesophageal reflux disease (GERD) without esophagitis**	Binary	4360 cases identified by secondary diagnostic codes of inpatient records and 458,650 controls from UK Biobank	9,851,867	HP ELISA titer [19]; CagA, VacA, UREA, Catalase, OMP, and GroEL antibody titers [22]	7/3/5/4/3/3/3 for HPELISA, CagA, VacA, UREA, Catalase, OMP, and GroEL	OR = 1.001 (*p*=0.043)/HP ELISA OR = 1.000(*p*=0.161)/CagA OR = 1.000 (*p*=0.732)/VacA OR = 1.000 (*p*=0.861)/UREA OR = 0.999 (*p*=0.467/Catalase OR = 0.999 (*p*=0.288)/OMP OR = 0.996 (*p*=0.773)/GroEL

Wang, 2024	2	**Irritable bowel syndrome (IBS)**	Binary	10,939 self-reported IBS cases and 451,994 controls from UK Biobank	9,851,867	HP ELISA titers [19]	10	OR = 1.00 (*p*=0.652)

Yang, 2024	2	**Inflammatory bowel disease (IBD)**	Binary	5673 cases identified by health administrative records/213,119 controls from FinnGen Biobank	16,380,466	HP seropositivity, VacA and GroEL antibody titers [22]	11 seropositivity 14 VacA 4 GroEL	OR = 1.19 (*p*=0.230)/HP positivity OR = 0.975 (*p*=0.309)/VacA OR = 1.016 (*p*=0.763)/GroEL
				12,882 cases based on symptoms and radiological, endoscopic, or histopathological findings/21,770 friend, spouse, or population controls from the participants in IBDGC consortium	12,716,084			

Zhu, 2024	2	**Eosinophilic esophagitis (EOE)**	Binary	1930 histologically confirmed pediatric cases and 13,634 controls from 4 consortia	11,310,926	HP seropositivity, CagA, Catalase, OMP, UreA, VacA, and GroEL antibody titers [22]	Seropositivity 7 CagA 2 Catalase 5 OMP 3 UreA 5 VacA 7 GroEL 2	Seropositivity OR = 0.325 (*p*=0.009) CagA OR = 0.937 (*p*=0.514) Catalase OR = 0.938 (*p*=0.463) OMP OR = 1.140(*p*=0.463) UreA OR = 1.021 (*p*=0.901) VacA OR = 0.942 (*p*=0.463) GroEL OR = 1.504 (*p*=0.181)

*Other health outcomes*								
Huang, 2024	2	**Miscarriage**	Binary	49,996 cases (by self-reported or electronic medical record) and 174,109 controls enrolled in global cohorts from Netherlands, Norway, United Kingdom, United States, and Australia	9,088,459	HP ELISA titers [19]	20	OR = 1.01 (*p*=0.712)
		**Preeclampsia-eclampsia**	Binary	3903 cases and 114,735 controls from FinnGen Biobank	16,379,723		18	OR = 1.12 (*p*=0.026)
		**Gestational diabetes mellitus**	Binary	5687 cases and 117,892 controls from FinnGen Biobank	16,379,784		20	OR = 0.98 (*p*=0.568)
		**Placental abruption**	Binary	294 cases and 104,247 controls from FinnGen Biobank	16,379,367		20	OR = 1.18 (*p*=0.371)
		**Premature rupture of membranes**	Binary	3011 cases and 104,247 controls from FinnGen Biobank	16,379,429		20	OR = 1.17(*p*=0.004)
		**Postpartum hemorrhage**	Binary	8249 cases and 202,621 controls from FinnGen Biobank	16,962,023		13	OR = 1.00 (*p*=0.947)
		**Neonate birthweight**	Continuous	210,267women from Early Growth Genetics (EGG) consortium and UK Biobank participants	14,869,762		19	OR = 0.99 (*p*=0.816)
		**Gestational age**	Continuous	84,689 women from EGG consortium and UK Biobank	7,646,297		18	OR = 0.99 (*p*=0.549)
		**Preterm birth**	Binary	4775 preterm births and 60,148 controls from EGG consortium	11,011,902		18	OR = 1.05(*p*=0.366)

Zhang, 2024c	2	**Primary open-angle glaucoma**	Binary	4433 cases/210,201 controls from FinnGen Biobank	16,380,455	HP ELISA titer [19]	66	OR = 1.00 (*p*=0.98)
		**Normal tension glaucoma**	Binary	892 cases/210,201 controls from FinnGen Biobank	16,380,455			OR = 0.97 (*p*=0.55)
		**Pseudo-exfoliation glaucoma**	Binary	1515 cases/210,201 controls from FinnGen Biobank	16,380,455			OR = 0.99 (*p*=0.77)

Jung, 2024	2	**IgA nephropathy (igAN)**	Binary	15,587 cases and 462,197 controls from United Kingdom and FinnGen Biobanks	24,182,646	HP seropositivity/binary, CagA, Catalase, GroEL, OMP, UreA, and VacA/continuous [22]	Seropositivity 13 CagA 20 Catalase 14 GroEL 9 OMP 15 UreA 22 VacA 23	Seropositivity OR = 0.843 (*p*=0.070) CagA OR = 0.999 (*p*=0.933) Catalase OR = 1.002 (*p*=0.951) GroEL OR = 1.003 (*p*=0.929) OMP OR = 0.969 (*p*=0.208) UreA OR = 0.998 (*p*=0.891) VacA OR = 1.027 (*p*=0.081)

Abbreviations: HbA1c, hemoglobin A1c; HDL, high-density lipoprotein; HP, *Helicobacter pylori*; LDL, low-density lipoprotein; OR, odds ratio; SD, standard deviation; SNP, Single-nucleotide polymorphism.

^∗^Boldface indicates health outcomes of primary interest, others mediators, or closely related conditions.

**Table 3:** Methodological details and authors' conclusion for the 16 Mendelian randomization studies of HP infection for various health outcomes.

Author, year	Criteria for IV SNP selection	No SNP selected	No SNP excluded	Test for independence assumption	Test for exclusion restriction	Additional analysis	Conclusion
den Hollander, 2017	*p* < 5 × 10^−8^, MAF > 1%, SNP with the lowest P from each loci	2	0	ND	ND	Adjusted for sex and age; bidirectional analysis	No causal relation between HP genetic risk score and BMI/obesity
Liu, 2022	Same as den Hollander 2017	2	1 for EPoS consortium and UK Biobank (not in the outcome GWAS)	ND	ND	Bidirectional analysis	No evidence for a causal link between HP infection and NAFLD
Zhang, 2024a	*p* < 5 × 10^−6^ excluding those in LD, with an *R*^2^ > 0.001, 10,000 kb window	12	0	ND	MR-Egger intercept, Cochran Q statistics	MR-Egger, weighted mode, simple mode, weighted median, and leave-one-out analysis for sensitivity analysis; bidirectional analysis	Study provides evidence for causal effects of *H. pylori* infection on osteoporosis
Sun, 2024	Same criteria as den Hollander 2017 for seropositivity; for others, *p* < 5 × 10^−5^, excluding those in LD with an *R*^2^ > 0.001, 10,000 kb distance and *p* < 5 × 10^−5^	2/90/80/68/86/88/93 for seropositivity/CagA/Catalase/GroEL/OMP/UreA/VacA	10/11/6/13/10/9 for CagA/Catalase/GroEL/OMP/UreA/VacA for T2DM; 53/5/50 GroEL SNP for FBS/Hb1/obesity due to absence in outcome data set	ND	MR-Egger intercept. Heterogeneity and pleiotropy tests	MR-Egger, weighted median, leave-one-out analysis, and MR-PRESSO for sensitivity analysis; analyses with instrumental SNPs for chronic gastritis and peptic ulcer; multivariable MR to adjust potential mediators	Our study found that *H. pylori* IgG antibody, *H. pylori* GroEL antibody, gastroduodenal ulcer, and chronic gastritis are all related to T2DM, and blood glucose level and obesity mediate the development of *H. pylori* GroEL antibody and gastroduodenal ulcer on T2DM
Li, 2024	Same as den Hollander 2018	2	1 for heart attack, stroke, arrythmia presumably due to absence in the outcome GWAS	ND	ND	Bidirectional analysis: Analysis for additional potential mediators, serum cytokines and vitamins, and total mortality	HP infection exerts a causal effect on CHD incidence, mediated by BMI
Wang, 2023	*p* < 5 × 10^−6^, excluding those with LD *r*^2^ ≥ 0.001	12	1 (outlier MS-PRESSO)	ND	MR-Egger intercept, MR-PRESSO	Leave-one-out analysis; analysis for additional potential mediators, i.e., pro- and anti-inflammatory cytokines, and vitamins C/D	Analysis revealed the causality of anti-H. pylori IgG levels on MI, which might be explained by lower HDL cholesterol levels
Zhang, 2024b	*p* < 5 × 10^−6^, LD clumping with an *R*^2^ < 0.001 and 10,000 kb, excluding outliers through radial MR, MR-PRESSO, and leave-one-out analyses	10/10/8/9/14 for HP seropositivity, CagA, Catalase, OMP, and VacA	2 HP seropositivity for cerebral AS, 3 VacA for coronary AS, 3 OMP for coronary and peripheral AS, 1 OMP for cerebral AS, and 5 OMP for other AS	ND	Cochran Q statistics, MR-PRESSO global test	MR-Egger, weighted median, and leave-one-out analysis for sensitivity analysis	MR study discovered a causal link between HP and AS. Different antibodies have different effects
Guo, 2023	*p* < 5 × 10^−6^, LD clumping with an *R*^2^ < 0.001 and 10,000 kb, removing those associated with confounders	11/8/10/7/4/11/15 for seropositivity OMP, UreA, Catalase, GroEL, CagA, and VacA	1–10 missing for specific antibodies and specific outcomes presumably due to absence in outcome GWAS	Removing SNPs with strong associations (undefined) with alcohol, smoking, BMI, diabetes, and educational level	MR-Egger intercept, Cochran Q statistics, MR-PRESSO global test	MR-Egger, weighted median, and leave-one-out analysis for sensitivity analysis, multivariable analyses with potential mediators. Gastroduodenal and peptic ulcers, CRP, MCP-1	Our results demonstrate that *H. pylori* VacA antibody is the only causal determinant associated with the risk of stroke in the spectrum of *H. pylori*–related antibodies, in which CRP may mediate the association
Luo, 2023	Same criteria as den Hollander 2017 for seropositivity; *p* < 5 × 10^−6^ for CagA and VacA, LD *r*^2^ < 0.001 or clump distance > 10,000 kb, F-statistics > 10, excluding SNPs with intermediate allele frequencies and ambiguous SNPs	2/15/15 for seropositivity/VacA/CagA	2 vacA and 5 cagA loci for CRC, and 1 vacA locus for colon and rectal cancer due to missing in respective outcome GWAS data	PhenoScanner search	MR_PRESSO and Cochran Q statistics	Bidirectional analysis, MR-Egger, and weighted median for sensitivity analysis for SNPs more than 2	No evidence for a causal association between seroprevalence of HP infection and colorectal cancer
Wang, 2024	*p* < 5 × 10^−6^	12	2 (strong LD, with an *R*^2^ < 0.001, a window size of 10,000 kb, and *p* < 5 × 10^−8^, intermediate allele frequencies or ambiguous SNPs with nonconcordant alleles)	ND	MR-Egger intercept, Cochran Q statistics	MR-Egger, weighted mode, weighted median, and leave-one-out analysis for sensitivity analysis	*H. pylori* infection may not be causally associated with an increased risk of IBS
Chen, 2023	*p* < 5 × 10^−6^, clumping with an *R*^2^ < 0.001 and 10,000 kb window, excluding those identified by PhenoScanner for confounders (*p* < 5 × 10^−8^) and those with F-statistics < 10	7/3/5/4/3/3/3 for HP ELISA, CagA, VacA, UREA, Catalase, OMP, and GroEL	0	PhenoScanner search for *p* < 5 × 10^−8^ associations	MR-Egger intercept, Cochran Q statistics	MR-Egger, weighted mode, simple mode, weighted median, and leave-one-out analysis for sensitivity analysis	Our two-sample Mendelian randomization analysis demonstrates a discernible link between the levels of HP IgG antibodies and an augmented susceptibility to GERD
Yang, 2024	*p* < 5 × 10^−6^ excluding those in LD clumping with an *R*^2^ < 0.001, 10,000 kb window and *p*-value < 5 × 10^−8^, SNPs with intermediate allele frequency, ambiguous SNPs	11/14/4 for seropositivity, VacA, and GroEL	1 HP-positivity SNP was missing in IBDGC	ND	Cochran Q statistics, MR-PRESSO global test	MR-Egger and weighted median, and bidirectional analysis	No direct correlation was observed between *H. pylori* infection and IBD risk
Zhu, 2024	*p* < 5 × 10^−6^, LD clumping with an *R*^2^ < 0.001 within 10,000 kb	7/4/5/4/5/8/3 for seropositivity, CagA, Catalase, OMP, UreA, VacA, and GroEL	Palindromic and ambiguous SNP, 1 each from CagA, OMP, VacA, and GroEL	SNPs associated with confounders (education attainment, household income, and deprivation), which potentially affect HP infection, were extracted from UK Biobank GWAS data. They were not removed but adjusted in multivariable analysis	MR-Egger intercept, Cochran Q statistics, MR-PRESSO global test	*p*-values adjusted with Benjamin–Hochberg correction for multiple comparisons. MR-Egger and weighted median estimates. Tests for addition mediators (circulating cytokines)	Findings suggested that among the range of *H. pylori*–related antibodies, anti-*H. pylori* IgG antibody is the sole causal factor associated with protection against EoE
Huang, 2024	*p* < 5 × 10^−5^ excluding those in LD, with an *R*^2^ > 0.001 with clumping window 10,000 kb, F-statistics < 10. MAF< 0.01 and palindromic SNPs	20	1, 2, or 7 excluded from some of the outcome presumably from absence in the outcome GWAS	ND	MR-Egger intercept, Cochran Q statistics, MR-PRESSO global test	MR-Egger, weighted mode, weighted median, and leave-one-out analysis for sensitivity analysis; bidirectional analysis	MR study demonstrated a causal relationship of HP infection with preeclampsia–eclampsia and premature rupture of membranes and confirmed the epidemiological evidence on the adverse impact of HP in pregnancy
Zhang, 2024c	*p* < 5 × 10^−5^, LD with an *R*^2^ < 0.001 with 10,000 kb window, F-statistics < 10, and palindromic SNPs	66	0	ND	MR-Egger intercept, Cochran Q statistics tests	Weighted median, weighted mode, simple mode, and leave-one-out analysis for sensitivity analysis; bidirectional analysis	These results indicated that there was no genetic evidence for a causal link between *H. pylori* and glaucoma
Jung, 2024	*p* < 5 × 10^−5^, LD clumping with an *R*^2^ < 0.001 within 10,000 kb, F-statistics > 10, excluding palindromic SNPs	17/24/16/9/16/24/24 for seropositivity, CagA, Catalase, Groel, OMP, UreA, and VacA	Exclusion due to associations with confounding factors 4/4/2/0/1/2/1 for seropositivity, CagA, Catalase, GroEL, OMP, UreA, and VacA	PhenoScanner and LDtrait search for SNPs associated with confounders, e.g., age, weight, hypertension, proteinuria, dental caries, periodontitis, tonsillitis, and other infections, including respiratory pathogens (e.g., Mycoplasma pneumoniae, herpes virus, influenza) and gut microbiota	MR-Egger intercept, Cochran Q statistics, MR-PRESSO global test	MR-Egger, weighted median, bidirectional analyses	There was no evidence of a causal relationship between *H. pylori* infection and IgAN

Abbreviations: GWAS, genome-wide association study; IV, instrumental variable, LD, linkage disequilibrium; MAF, minor allele frequency; ND, not done.

**Table 4:** Summary of functional mapping and annotation analysis for 89 SNPs with *p* values 5 × 10^−6^ reported in supplementary data of 16 MR studies.

Genomic locus	Antibody	rsID	chr	Position	Reference allele	Alternative allele	MAF in European	GWAS P	Beta	Se	Nearest gene	Distance (bp)	SNP type	CADD	eQTL *p* value	eQTL gene
1	VacA	rs72645538	1	9,453,174	A	G	0.0159	0.00000466	0.75279	0.16438	SPSB1	23,582	Intergenic	3.306		
2	Positivity	rs60696037	1	23,082,564	C	T	0.1074	0.00000199	−0.05611	0.0118	EPHB2	0	Intronic	5.952		
3	Titer	rs41263973	1	32,162,810	G	A	0.03479	0.00000274	0.31578	0.06723	COL16A1	0	Intronic	0.335		
4	OMP	rs703135	1	158,678,095	G	A	0.4225	0.00000209	−0.13318	0.02807	OR6K2	7608	Intergenic	6.893		
5	Upper25%	rs368433	1	161,484,210	T	C	0.16	0.000000021	−0.31481	0.0561	FCGR2A	0	Intronic	—	9.5E − 19/2.5E − 20	FCGR2B
6	CagA	rs116421363	1	181,820,561	A	C	0.3688	0.00000268	−0.22335	0.04758	RN7SKP229	11,651	Intergenic	0.051		
7	VacA	rs113063793	1	217,309,978	A	T	0.04771	0.000000797	0.3734	0.07565	ESRRG	0	Intronic	6.261		
8	OMP	rs55862931	2	2,672,878	G	C	0.1039	0.00000382	−0.14634	0.03167	AC018685.1	12,475	Intergenic	1.958		
9	Catalase	rs58679787	2	16,452,055	C	T	0.02087	0.00000384	0.77652	0.16808	AC010745.2	41,646	Intergenic	2.102		
10	Titer	rs2169557	2	20,244,954	C	T	0.493	0.00000455	−0.10591	0.02307	LAPTM4A	0	Intronic	10.14		
11	OMP	rs143477841	2	24,042,805	G	A	0.0169	0.00000437	−0.57253	0.12466	ATAD2B	0	Intronic	2.437		
12	OMP	rs1156822	2	41,824,828	A	T	0.4264	0.00000436	−0.12761	0.02778	AC010739.1	1195	Intergenic	4.983		
13	Catalase	rs72808426	2	58,133,048	C	T	0.0328	0.00000379	−0.43391	0.09386	VRK2	1737	Intergenic	0.596		
14	Positivity	rs681900	2	75,074,967	T	C	0.2167	0.00000212	−0.0375	0.00791	HK2	0	Intronic	15.19		
15	Catalase	rs190890483	2	157,083,346	G	A	0.01789	0.000000863	−0.7718	0.15685	NR4A2	97,597	Intergenic	2.681		
16	CagA	rs75170215	3	25,908,239	T	C	0.02684	0.00000202	0.74326	0.15642	LINC00692	0	Intronic	1.27		
17	VacA	rs113845906	3	94,372,336	G	A	0.02485	0.000000997	0.41348	0.08452	ARMC10P1	145,871	Intergenic	0.041		
18	UreA	rs9289888	3	152,849,257	C	T	0.07952	0.00000212	0.27208	0.05738	RP11-529G21.3	1827	Intergenic	0.59	0.0000111726	ARHGEF26-AS1
19	UreA	rs4689912	4	4,659,633	T	C	0.2575	0.0000019	−0.15801	0.03317	STX18-AS1	0	ncRNA_intronic	3.284		
20	Upper25%	rs10004195	4	38,784,724	T	A	0.25	1.4E-18	−0.35767	0.04	TRL10	113	Upstream	—	2.1E − 04/3.2E − 17	TLR1
21	Titer	rs77516628	4	62,125,618	A	T	0.07356	0.00000294	0.18787	0.04013	LPHN3	0	Intronic	16.46		
22	CagA	rs75740599	4	159,123,611	G	A	0.2495	0.00000427	0.23584	0.0513	RP11-597D13.9:TMEM144	1	ncRNA_exonic	1.049		
23	Titer	rs72708546	4	170,153,219	G	A	0.05268	0.0000019	−0.22969	0.04814	SH3RF1	0	Intronic	0.891		
24	Positivity	rs10044474	5	67,425,603	T	G	0.08847	0.00000185	0.06539	0.01371	RNU6-1232P	29,285	Intergenic	3.274		
25	VacA	rs1530121	5	124,624,902	T	C	0.161	0.000000196	−0.26611	0.05114	HMGB1P22	23,557	Intergenic	3.607		
26	UreA	rs71569678	6	23,380,648	A	C	0.06163	0.0000000299	0.34017	0.06138	RP11-439H9.1	33,859	Intergenic	3.973		
27	Catalase	rs6456714	6	26,335,346	C	G	0.3549	0.00000218	0.1709	0.03609	HIST1H3PS1	12,825	Intergenic	0.266		
28	VacA	rs372744619	6	32,434,141	A	G	0.01889	0.000000329	0.88741	0.1738	HLA-DRB9	0	ncRNA_intronic	5.266		
28	CagA	rs3998182	6	32,561,915	T	C	0.2306	0.000000204	0.28476	0.0548	HLA-DRB1	4289	Intergenic	9.045	0.00000000297749	HLA-DQB1
28	OMP	rs3104361	6	32,652,278	T	C	0.4384	6.48E-10	0.18149	0.02938	HLA-DQB1	16,117	Intergenic	1.357	0.00000000835393	HLA-DQB1
28	Titer	rs35030589	6	32,672,903	G	A	0.1948	0.00000034	−0.17516	0.03429	MTCO3P1	997	Downstream	3.619	0.0000246373	TAP2
28	OMP	rs9276733	6	32,766,026	A	G	0.335	0.0000000814	0.1957	0.03648	HLA-DOB	14,513	Intergenic	3.816	0.0000695545	HLA-DRB5
29	Catalase	rs343686	6	80,607,062	A	C	0.3917	0.00000175	−0.1747	0.03655	ELOVL4	17,466	Intergenic	0.69		
30	GroEL	rs3104037	6	107,139,657	C	T	0.3638	0.0000019	0.13414	0.02816	RP1-60O19.2	3752	Intergenic	1.646		
31	CagA	rs571061419	6	162,760,485	G	A	0.08052	0.00000421	−0.39214	0.08524	PARK2	0	Intronic	1.889		
32	Titer	rs117912702	6	166,062,930	G	A	0.02584	0.00000302	0.40503	0.0866	PDE10A	0	Intronic	3.614		
33	VacA	rs10246445	7	2,981,123	A	C	0.02187	0.00000492	−0.48814	0.10686	CARD11	0	Intronic	3.467		
34	VacA	rs77497849	7	32,049,959	A	G	0.1451	0.000000913	0.26423	0.05382	PDE1C	0	Intronic	4.373		
35	Positivity	rs117192929	7	89,122,359	A	G	0.03777	0.0000043	0.07672	0.01669	AC002383.2	0	ncRNA_intronic	11.51		
36	Catalase	rs77162569	7	148,264,987	C	T	0.03181	0.000000327	−0.55554	0.10878	AC005229.5	12,505	Intergenic	4.845		
37	CagA	rs6530847	8	15,111,565	T	A	0.3598	0.00000341	−0.21027	0.04527	SGCZ	15,716	Intergenic	6.12		
38	OMP	rs60949128	8	41,936,404	C	A	0.07058	0.000000853	−0.2773	0.05633	RP11-589C21.1	22,431	Intergenic	1.212		
39	UreA	rs75477465	8	68,794,331	G	A	0.03976	0.00000303	0.37487	0.0803	NDUFS5P6	32,757	Intergenic	4.657		
40	OMP	rs116944686	8	101,374,808	G	A	0.01789	0.00000478	0.41781	0.09134	KB-1991G8.1	11,872	Intergenic	0.263		
41	Positivity	rs17232730	8	129,537,746	G	C	0.1034	0.000000691	−0.05211	0.0105	RP11-89M16.1	0	ncRNA_intronic	0.284		
42	VacA	rs7019543	9	110,365,310	G	T	0.333	0.00000201	0.18107	0.0381	RP11-438P9.2	59,412	Intergenic	2.552		
43	Positivity	rs111575036	10	45,194,547	C	T	0.02485	0.000004	−0.11655	0.02527	RP11-733D4.1	96,994	Intergenic	1.956		
44	cagA	rs4268452	10	81,319,354	C	T	0.07256	0.00000462	−0.38498	0.08403	SFTPA2	0	UTR5	1.566	0.0000343656	NUTM2B
45	Positivity	rs11201988	10	88,113,475	G	A	0.1153	0.0000038	0.05057	0.01094	GRID1	0	Intronic	4.115		
46	VacA	rs117077218	10	90,083,333	C	T	0.0159	0.00000395	0.64022	0.13876	RNLS	0	Intronic	10.4		
47	GroEL	rs61680606	10	128,939,945	A	C	0.2594	0.00000238	0.15227	0.03227	DOCK1:FAM196A	1	Intronic	0.679		
47	VacA	rs59104649	10	129,150,690	A	T	0.02087	0.00000344	0.68347	0.14721	DOCK1	0	Intronic	0.17		
48	GroEL	rs7099832	10	130,790,371	G	C	0.4901	0.00000131	−0.1338	0.02766	RP11-442O18.1	32,044	Intergenic	0.237		
49	CagA	rs138363822	11	1,606,147	A	G	0.4563	0.0000043	0.21833	0.0475	KRTAP5-AS1:KRTAP5-1	1	Exonic	5.701		
50	Titer	rs73512476	11	86,585,900	G	T	0.07058	0.00000147	0.21258	0.04408	PRSS23	0	Intronic	1.56		
51	CagA	rs117537486	11	94,351,805	G	C	0.03082	0.00000422	0.59679	0.12974	PIWIL4:RP11-867G2.8	1	ncRNA_intronic	2.389	0.0000148341	RP11-685N10.1
52	CagA	rs117827497	11	105,212,623	A	G	0.02485	0.0000031	−0.58373	0.12515	RP11-94P11.4	0	ncRNA_intronic	2.204		
53	UreA	rs61907106	12	4,003,258	G	A	0.0338	0.000000327	−0.35885	0.07026	RP11-664D1.1	0	ncRNA_intronic	4.149		
54	VacA	rs11044935	12	20,062,085	A	T	0.04374	0.00000222	0.42828	0.09051	RP11-405A12.2	0	ncRNA_intronic	5.033		
55	VacA	rs148556020	12	28,904,914	T	A	0.01889	0.00000444	0.63085	0.13746	RP11-977P2.1	69,993	Intergenic	0.33		
56	Positivity	rs74347185	12	75,543,807	G	A	0.04175	0.000000253	0.07804	0.01514	KCNC2	0	Intronic	0.909		
57	CagA	rs149747348	12	98,890,443	G	C	0.0169	0.00000392	−0.89541	0.194	RP11-181C3.1	0	Intronic	2.078		
58	UreA	rs12820262	12	129,244,638	G	A	0.165	0.000000159	0.20607	0.03932	SLC15A4	33,100	Intergenic	1.232		
59	Titer	rs17502937	13	30,440,740	G	T	0.03181	0.00000289	−0.39671	0.08466	TIMM8BP1	433	Upstream	0.01		
60	Catalase	rs17647677	13	46,749,781	T	C	0.04672	0.00000365	−0.39412	0.08512	LCP1	0	Intronic	6.312		
61	GroEL	rs117341694	13	66,889,675	G	A	0.01789	0.00000273	0.54338	0.11586	PCDH9:PCDH9-AS1	1	ncRNA_intronic	0.218		
62	CagA	rs56264437	13	102,123,887	G	A	0.3559	0.00000199	0.2339	0.0492	ITGBL1	0	Intronic	0.025		
63	Catalase	rs927062	14	33,095,049	A	G	0.1869	0.0000026	−0.19794	0.04211	AKAP6	0	Intronic	8.937		
64	Titer	rs74045808	14	34,457,396	C	T	0.1093	0.00000449	−0.17464	0.03801	EGLN3	0	Intronic	0.718		
65	OMP	rs8019346	14	65,353,011	G	A	0.09245	0.00000424	0.22927	0.04985	SPTB	6409	Intergenic	1.329		
66	UreA	rs60386004	14	80,136,052	A	T	0.1928	0.00000249	0.19177	0.04073	NRXN3:RP11-242P2.1	1	ncRNA_intronic	0.525		
67	Positivity	rs76753623	15	61,751,677	A	G	0.04175	0.000000976	0.08427	0.01721	RP11-507B12.2	0	ncRNA_intronic	8.174		
68	Titer	rs55871438	15	75,411,120	T	C	0.04771	0.00000402	0.29919	0.0648	PPCDC	1316	Intergenic	10.14		
69	Titer	rs78825412	15	83,512,621	C	A	0.0328	0.00000364	0.31781	0.06852	HOMER2	0	UTR3	0.205		
70	CagA	rs11858369	15	90,495,509	A	G	0.07952	0.000000418	0.43772	0.0865	RP11-493E3.2	15,271	Intergenic	2.159		
71	Titer	rs12591869	15	96,674,267	C	A	0.2346	0.00000129	−0.1286	0.02651	NR2F2-AS1	0	ncRNA_intronic	0.612		
72	VacA	rs73500239	16	1,332,341	G	C	0.1481	0.00000238	0.23609	0.05004	RP11-616M22.9	1795	Intergenic	0.808		
73	CagA	rs118006294	16	10,974,221	T	C	0.03082	0.00000304	−0.4753	0.10182	RP11-876N24.2:CIITA	1	ncRNA_intronic	3.564		
74	Catalase	rs147016571	16	25,837,192	A	G	0.01292	0.00000176	−0.83995	0.17576	HS3ST4:RP11-185O21.1	1	ncRNA_exonic	7.016		
75	CagA	rs553266653	16	46,420,007	A	C	0.09443	0.00000404	−0.73151	0.15871	ANKRD26P1	83,245	Intergenic	9.001		
76	Positivity	rs9747624	17	1,022,796	G	C	0.02982	0.00000289	0.08388	0.01793	ABR	0	Intronic	4.481		
77	UreA	rs143570118	17	76,370,080	C	T	0.02286	0.00000276	0.43413	0.0926	RP11-806H10.4	87	Downstream	3.776		
78	GroEL	rs1367344	18	71,479,072	T	C	0.2217	0.00000268	−0.1552	0.03306	RN7SL401P	66,607	Intergenic	0.525		
79	UreA	rs117994655	19	8,188,056	G	A	0.04076	0.00000121	0.40202	0.08283	FBN3	0	Intronic	1.176		
80	Positivity	rs62195865	20	8,239,626	T	C	0.01193	0.00000223	0.11718	0.02477	PLCB1	0	Intronic	1.474		
81	OMP	rs1892331	20	59,326,926	A	G	0.02087	0.00000074	0.37696	0.07615	RP11-151E14.1	60,133	Intergenic	0.829		
82	VacA	rs9606224	22	20,034,187	C	T	0.02684	0.000000691	0.50746	0.10223	TANGO2	0	Intronic	2.989		
83	UreA	rs134748	22	26,543,287	G	T	0.33	0.0000035	−0.1422	0.03065	CTA-796E4.3	14,512	Intergenic	0.16		
84	VacA	rs133537	22	48,595,971	T	C	0.3191	0.00000225	−0.17361	0.03671	LL22NC03-121E8.3	56,924	Intergenic	1.826		

*Note:* Antibody: CagA, VacA, GroEL, UreA, and OMP. Catalase, positivity from Butler–Laporte, 2020; titer from Chong, 2021; upper 25% from Mayerle, 2013 (see Table 1), CDD: Measure of SNP deleteriousness. Higher score is more likely to be deleterious; eQTL was tested for stomach, except rs368433 and rs10004195 that were tested for blood.

Abbreviation: SE, standard error.

## Data Availability

Data sharing is not applicable to this article as no new data were created or analyzed in this study.
